# Translation of the Chinese Version of the Nomophobia Questionnaire and Its Validation Among College Students: Factor Analysis

**DOI:** 10.2196/13561

**Published:** 2020-03-13

**Authors:** Ye Gao, Hongliang Dai, Guizhi Jia, Chunguang Liang, Tong Tong, Zhiyu Zhang, Ruobing Song, Qing Wang, Yue Zhu

**Affiliations:** 1 School of Nursing Jinzhou Medical University Jinzhou China; 2 Department of Physiology Jinzhou Medical University Jinzhou China; 3 Department of Physiology Shanxi Medical University Taiyuan China

**Keywords:** nomophobia, reliability, validity, mobile phone

## Abstract

**Background:**

Nomophobia or phobia of no mobile phone is the fear of being without a mobile phone or being unable to contact others via a mobile phone. It is a newly emerging psychiatric disorder among mobile phone users.

**Objective:**

There are no psychometric scales available in China for examining nomophobia, although China has become the largest mobile phone handset consumer market in the world. Therefore, this study aimed to translate the original English version of a psychometric scale into Chinese and further examine its reliability and validity among Chinese college students.

**Methods:**

The original version of the Nomophobia Questionnaire (NMP-Q) was first translated into Chinese using the backward and forward translation procedure. An exploratory factor analysis (a principal component analysis plus varimax rotation) and a confirmatory factor analysis (CFA) were performed to examine the underlying factor structure of the translated questionnaire. The internal consistency reliability of the scale was determined by computing the Cronbach alpha coefficient, the test-retest reliability, and the corrected item-total correlation. A multivariate regression analysis was used for examining associations between nomophobia and independent variables among the college students.

**Results:**

A total of 2000 participants were included in the study. Their ages ranged from 16 to 25 years, with 51.95% (1039/2000) being male participants. The Chinese version of NMP-Q retained 18 items. The eigenvalues, total variance explained, and scree plot jointly support a 4-factor structure of the translated questionnaire. The CFA reached the adaptive standard, and the discriminant validity of the scale was good. The Cronbach alpha coefficient of this scale was .925, and the Cronbach alpha coefficients of the subscales were .882, .843, .895, and .818. The test-retest reliability was 0.947. Corrected item-total correlation ranged from 0.539 to 0.663. The significant predictors for each of the dimensions of nomophobia and total score of the questionnaire were the average number of hours spent on a mobile phone daily and gender.

**Conclusions:**

The Chinese version of the NMP-Q exhibited satisfactory psychometric properties.

## Introduction

### Background

The mobile phone embodies the latest evolution of modern information and communication technologies [1]; in recent years, it has become increasingly widespread within the Chinese society. In fact, China has become the largest mobile phone handset consumer market in the world, and the market is expected to grow rapidly in the coming years [2]. As of December 2016, there were 695 million mobile phone subscribers with access to the internet in China, according to China’s 39th Statistical Report on Internet Development [2]. Indeed, in addition to the traditional *phone* function for the purpose of communication, mobile phones nowadays are more akin to powerful portable computers, providing a number of other intellectual functions such as internet browser, camera, email services, real-time information, social networking, and personal diary. All these powerful functions of the mobile phone make our life unprecedentedly more dynamic, effective, and convenient, thereby making the use of mobile phones increasingly widespread among individuals from different countries and regions [1-3].

Despite the countless benefits associated with mobile phone use, it is severely damaging to our health, both physically and mentally. Previous studies suggest that mobile phone overuse exists among many people, which seriously interferes with their daily lives, safety, and health status. These health-related problems include blurred vision, pain in the wrists or neck, “screen dermatitis,” tumors, infertility, and, in many cases, traffic accidents [4-6]. Moreover, smartphone overuse also harms mental health, leading to “smartphone dependence or addiction” [2,7,8], which is characterized by compulsive phone use, intolerance, withdrawal, and functional impairment. Other terms describing mobile phone–related mental disorders include “SMS texting addiction,” “compulsive selfie-taking behavior,” “sexting,” “phubbing,” and the recently emerged “nomophobia” [1,9-11].

Nomophobia, the contraction of no mobile phone phobia, is a recently emerging neologism to describe the anxiety and distress among mobile phone users when they are without a smartphone or mobile phone and are unable to get access to the services and real-time information it provides and feel disconnected. The characterized symptoms of nomophobia include overuse of a mobile phone and the resultant feeling of disturbance when there is a lack of network coverage, the battery is out of power, or the balance between the two is not enough. In addition, people with nomophobia would frequently check their phones for messages or missed calls, and they are repeatedly under the illusion of hearing a mobile phone ring or vibrate. All these symptoms because of the problematic use of a mobile phone would inevitably affect people’s social life, work productivity, and academic performance in a negative way. To evaluate this situational phobia status affecting mobile phone users, Yildirim and Correia [12] first developed the Nomophobia Questionnaire (NMP-Q) in the United States in the English language as an effective instrument. To the best of our knowledge, since the advent of the English version of NMP-Q, it has only been translated into 4 other languages, Italian [1], Persian [13], Spanish [14], and most recently Chinese [15], with proven reliability in each version. In 2018, Jianling Ma and Chang Liu translated the English version of NMP-Q based on the cultural background and language habits of the Chinese and explored the applicability of the Chinese version of the scale among 966 college students in southern China, and the results showed that the reliability and validity were good. However, whether the NMP-Q scale can be directly used to evaluate the level of nomophobia of college students in northern China needs to be confirmed.

### Objectives

The aims of this study were to translate the original NMP-Q into simplified Chinese and further confirm its reliability and validity among college students in northern China.

## Methods

### Instrument

NMP-Q is a 20-item scale developed by Yildirim and Correia [12] through a thorough procedure comprising qualitative and quantitative phases. NMP-Q comprises 4 factors (Factor 1: losing connectedness; Factor 2: giving up convenience; Factor 3: not being able to communicate; Factor 4: not being able to access information). A 7-point Likert scale ranging from 1 (strongly disagree) to 7 (strongly agree) is applied to each NMP-Q item, leading to a summed total score. After obtaining permission from the original authors, the Chinese NMP-Q was developed by using the original English version and the classical “backward and forward” procedure [1]. The final Chinese NMP-Q (Multimedia Appendix 1) was then administered to 2000 college students in their universities in Jinzhou, along with a general questionnaire to record the variables of age, gender, whether having a girlfriend or boyfriend, average daily hours of mobile phone use, residence, and grade.

### Participants and Procedure

This was a cross-sectional study, and the participants comprised college students aged 16 to 25 years from 3 universities in Jinzhou, China. The participants who possessed a mobile phone were recruited in this study via a convenience sampling method. The research procedures complied with the ethical standards of the institutional research committee, as well as the 1964 Helsinki declaration and its later amendments. All these students had been given written informed consent.

### Statistical Analysis

Statistical analysis was performed using SPSS 22.0 and AMOS 21.0 (IBM Corporation). Continuous data were presented as mean (SD), whereas categorical data were expressed as percentages. Skewness and kurtosis were computed for each item, and the data were considered normally distributed when the values ranged from -2 to +2 [1]. To investigate the underlying factor structure of the translated questionnaire, an exploratory factor analysis (EFA) and a confirmatory factor analysis (CFA) were performed. The sample of 2000 cases was randomly divided into 2 groups, one (n=1022) for EFA and the other (n=978) for CFA. In EFA (n=1022), a principal component analysis (PCA) with varimax rotation was performed on the 20 items of the questionnaire. Varimax rotation is the most commonly used orthogonal technique that minimizes factor complexity with a maximized variance of factor loading. The sampling adequacy for the factorability was assessed using the Kaiser-Meyer-Olkin (KMO) [16] measurement and Bartlett test [17] of sphericity. Only when the Bartlett test of sphericity was significant (*P*<.05) and the KMO was >0.60, the dataset was considered appropriate for PCA. The factors were extracted based on the comprehensive consideration of eigenvalues, explained total variance, and a visual inspection of the scree plot. AMOS (IBM Corporation) was used to perform the CFAs of NMP-Q, analyzing the fit of models and its respective parameter estimates.

Discriminant validity is the total score of the NMP-Q scale, which was ranked from high to low; the top 27% of the scores were grouped into the high-score group, the bottom 27% of the scores were grouped into the low-score group, and the scores of each item in the 2 groups were analyzed by using a two-tailed independent samples *t* test. If the scores of each item in the 2 groups reached the significance level (*P*<.05), discriminant validity was considered good.

**Figure 1:**
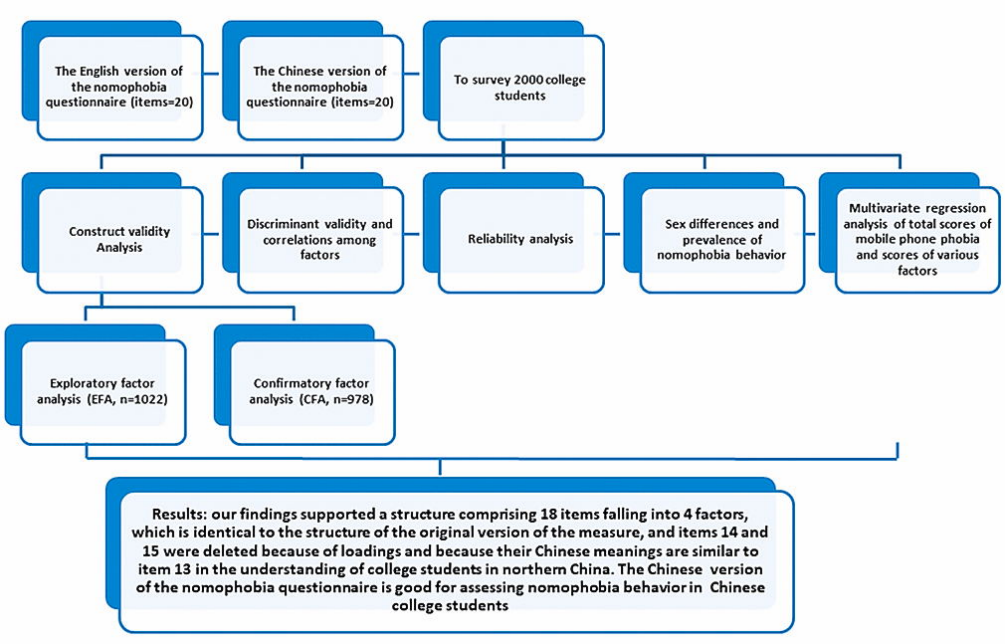
Diagram showing the flow of the study.

Reliability for internal consistency of the scale was examined by calculating the Cronbach alpha coefficient, the test-retest reliability, and the corrected item-total correlation. The minimum acceptable Cronbach alpha coefficient was set at .7 [18]. The corrected item-total correlation, representing the correlation between each item and the sum of the other items in a scale, was performed using the standard of 0.3 for inclusion [18,19]. A multivariate regression analysis was performed to explore the underlying independent variables of nomophobia. The flow of the study is presented in Figure 1.

## Results

### Descriptive Statistics

This investigation was conducted among 2000 college students, with ages ranging from 16 to 25 years. A total of 51.95% (1039/2000) of participants were males. Regarding daily mobile phone use, 5.70% (114/2000) of participants usually spent less than 2 hours on their mobile phones, 25.35% (507/2000) of participants spent between 2 and 4 hours, 42.40% (848/2000) of participants spent between 4 and 6 hours, 13.85% (277/2000) of participants spent between 6 and 8 hours, 6.65% (133/2000) of participants spent between 8 and 10 hours, and 6.05% (121/2000) of participants spent more than 10 hours.

The mean (SD) score of each item of the Chinese NMP-Q is shown in Table 1. These data were normally distributed according to the skewness and kurtosis figures.

**Table 1:** Mean (SD) scores with skewness and kurtosis figures (N=2000).

Item number	Mean (SD)	Skewness	Kurtosis
1	3.61 (1.785)	0.193	-1.025
2	3.57 (1.787)	0.215	-1.078
3	3.10 (1.636)	0.539	-0.616
4	4.04 (1.808)	-0.178	-1.127
5	4.28 (1.907)	-0.311	-1.146
6	4.14 (1.882)	-0.214	-1.169
7	4.11 (1.958)	-0.158	-1.284
8	4.24 (1.850)	-0.249	-1.125
9	4.47 (1.737)	-0.470	-0.785
10	4.74 (1.761)	-0.653	-0.617
11	4.75 (1.764)	-0.660	-0.597
12	4.62 (1.763)	-0.563	-0.723
13	4.17 (1.791)	-0.178	-1.027
14	3.96 (1.785)	-0.061	-1.076
15	4.01 (1.794)	-0.102	-1.055
16	3.63 (1.772)	0.192	-0.992
17	3.24 (1.711)	0.440	-0.757
18	3.51 (1.778)	0.226	-1.022
19	2.87 (1.649)	0.713	-0.332
20	3.45 (1.828)	0.248	-1.045

### Construct Validity Analysis

Before commencing an EFA, the factorability of the matrix of a sample (n=1022) was first examined. The Bartlett test [17] of sphericity was significant (χ^2^_190_=12,413.0; *P*<.001), and the KMO index [16] was 0.934, which is greater than the minimum acceptable value of 0.6. Therefore, the matrix is not an identity matrix and is appropriate for factor extraction.

It was shown that the vast majority of the items (16/20) were loaded on a single factor, and the loadings on other factors were much lower. The loading of items 4, 14, 15, and 20 was greater than 0.4 on 2 different factors [20]. After a discussion with experts, items 14 and 15 were deleted as they were similar to item 13 in Chinese. For college students in northern China, items 4 and 20 were reserved because of their influence and contribution rate on the scale structure. A re-exploratory factor analysis was performed after deletion; the Bartlett test [17] of sphericity was significant (χ^2^_153_=10,609.0; *P*<.001), and the KMO index [16] was 0.928. The results are shown in Table 2.

**Table 2:** Factor loadings of the Nomophobia Questionnaire (N=1022; salient factor loadings are indicated in italics).

Item number	Factor 1	Factor 2	Factor 3	Factor 4
17	*0.846*	0.123	0.133	0.235
19	*0.834*	0.051	0.092	0.163
18	*0.800*	0.255	0.109	0.154
16	*0.746*	0.257	0.211	0.136
20	*0.584*	0.452	0.108	0.079
6	0.163	*0.800*	0.104	0.248
5	0.121	*0.753*	0.187	0.314
9	0.233	*0.679*	0.287	0.118
7	0.239	*0.655*	0.180	0.179
8	0.189	*0.564*	0.372	0.209
11	0.086	0.193	*0.889*	0.104
12	0.131	0.172	*0.850*	0.116
10	0.086	0.307	*0.817*	0.110
13	0.291	0.116	*0.673*	0.278
2	0.139	0.292	0.160	*0.770*
3	0.385	0.078	0.151	*0.754*
4	0.070	0.481	0.153	*0.646*
1	0.227	0.342	0.181	*0.586*

**Figure 2:**
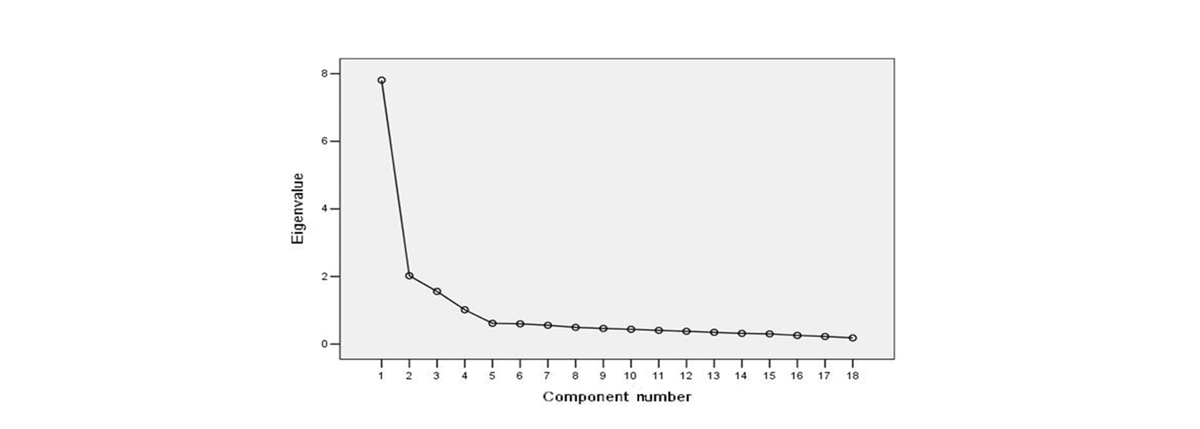
Screen plot of exploratory factor analysis for Chinese version of the Nomophobia Questionnaire.

**Figure 3:**
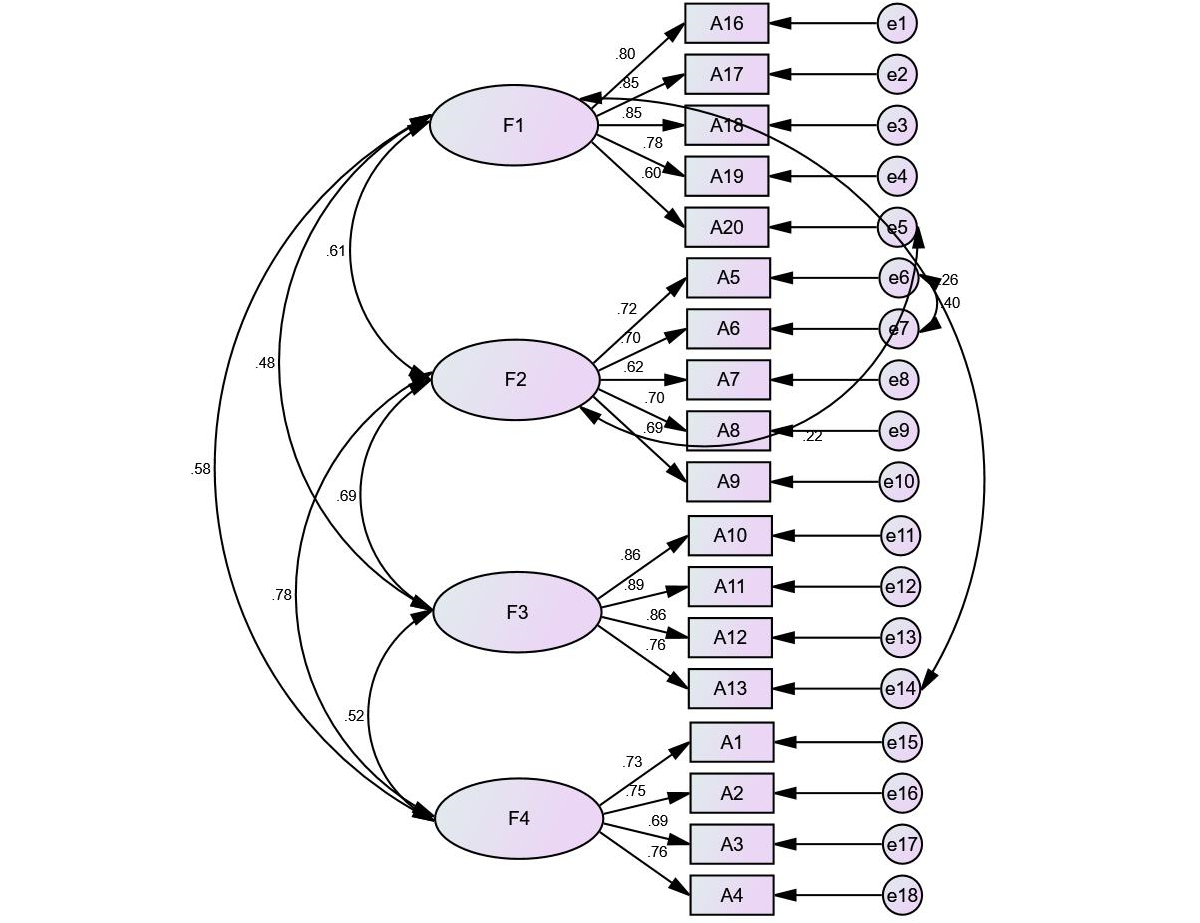
Standardized four-factor structural model of the Nomophobia Questionnaire (n=978). F1 (losing connectedness, five items), F2 (giving up convenience, five items), F3 (not being able to communicate, four items), F4 (not being able to access information, four items).

The first PCA was run to determine the likely number of factors. As a result, 4 factors that explained a total of 68.93% of the variance had initial eigenvalues >1 each. The 4-factor structure was further confirmed by the scree plot, as the descending tendency became weak after the fourth point. After varimax rotation, these 4 extracted factors explained 43.39%, 11.24%, 8.66%, and 5.65% of the variance. The scree plot is shown in Figure 2. A CFA was performed on the sample (n=978). In the model fitness index, the chi-square degree of freedom was 4.967, goodness-of-fit index was 0.933, adjusted goodness-of-fit index was 0.909, parsimonious goodness-of-fit index was 0.687, incremental fit index was 0.952, Tucker Lewis index was 0.942, comparative fit index was 0.952, root mean square error of approximation was 0.064, and standardized root mean residual was 0.049. The CFA results are shown in Figure 3.

### Discriminant Validity and Correlations Among Factors

The total score of the NMP-Q scale was ranked from high to low; the top 27% of the scores were grouped into the high-score group, and the bottom 27% of the scores were grouped into the low-score group. In this study, critical value scores were 83 and 58, respectively, and the score of each item in the 2 groups was analyzed by using a 2-tailed independent samples *t* test. The results showed that the score of each item in the 2 groups reached the level of significance (*P*<.05). The results are shown in Table 3. The results of correlations among factors in the Chinese version of NMP-Q are shown in Table 4.

**Table 3:** Score comparison between high-score and low-score groups (N=2000).

Item	Low-score group (n=553), mean (SD)	High-score group (n=567), mean (SD)	*t* test (*df*)	*P* value
1	2.21 (1.286)	5.07 (1.444)	-35.077 (1108.951)	<.001
2	2.22 (1.319)	5.00 (1.480)	-33.217 (1109.057)	<.001
3	1.90 (1.024)	4.35 (1.596)	-30.634 (967.443)	<.001
4	2.52 (1.514)	5.51 (1.165)	-36.917 (1036.226)	<.001
5	2.52 (1.536)	5.86 (1.099)	-41.765 (998.128)	<.001
6	2.45 (1.479)	5.64 (1.274)	-38.591 (1085.682)	<.001
7	2.58 (1.584)	5.63 (1.351)	-34.609 (1081.911)	<.001
8	2.62 (1.521)	5.71 (1.115)	-38.609 (1011.067)	<.001
9	2.98 (1.673)	5.76 (1.044)	-33.304 (921.158)	<.001
10	3.21 (1.802)	6.00 (0.929)	-32.446 (821.716)	<.001
11	3.29 (1.866)	5.98 (0.959)	-30.136 (819.922)	<.001
12	3.18 (1.759)	5.85 (1.093)	-30.476 (918.955)	<.001
13	2.61 (1.474)	5.57 (1.216)	-36.585 (1068.760)	<.001
16	2.20 (1.319)	5.15 (1.412)	-36.109 (1115.903)	<.001
17	1.94 (1.125)	4.65 (1.525)	-33.970 (1041.411)	<.001
18	2.05 (1.213)	4.97 (1.415)	-37.104 (1100.063)	<.001
19	1.80 (1.000)	4.02 (1.743)	-26.223 (906.649)	<.001
20	2.06 (1.245)	4.86 (1.629)	-32.421 (1057.658)	<.001

**Table 4:** Correlations among factors in the Chinese version of the Nomophobia Questionnaire (N=2000).

Factor	Factor 1	Factor 2	Factor 3
Factor 2	0.556^a^	—^b^	—
Factor 3	0.443^a^	0.569^a^	—
Factor 4	0.542^a^	0.663^a^	0.465^a^

^a^Significant correlation at the .01 level (two-sided).

^b^Not available.

### Reliability Analysis

Reliability analysis results showed that the Chinese version of NMP-Q has ideal internal consistency, with the overall Cronbach alpha coefficient being .925 and Cronbach alpha coefficients for the 4 factors being .882, .843, .895, and .818, all greater than the minimum acceptable value of .7. The change in the Cronbach alpha value when a given item is excluded from the questionnaire is listed in Table 5. As seen in Table 5, the deletion of each item each time exclusively led to a decrease in the Cronbach alpha coefficient of the questionnaire (.925). In addition, the corrected item-total correlation of the items ranged from 0.539 to 0.663, that is, all greater than 0.3. As such, 18 items are integral to the questionnaire. After 2 weeks, 30 students were randomly selected for retesting; the test-retest reliability was 0.947.

**Table 5:** Cronbach alpha coefficient if the item was deleted and corrected item-total correlation (N=2000).

Item	Cronbach alpha if the item was deleted	Corrected item-total correlation
1	.921	0.600
2	.922	0.585
3	.922	0.577
4	.921	0.632
5	.920	0.663
6	.920	0.649
7	.922	0.586
8	.921	0.637
9	.921	0.620
10	.921	0.617
11	.922	0.588
12	.922	0.592
13	.921	0.636
16	.920	0.652
17	.921	0.622
18	.921	0.634
19	.923	0.539
20	.922	0.596

### Sex Differences and Prevalence of Nomophobic Behavior

The independent *t* test found that females (mean 75.61, SD 20.32) had a higher total nomophobia score than males (mean 65.82, SD 21.23; t_2000_= -10.52; *P*<.001). To evaluate the prevalence of nomophobic behavior in Chinese college students, a total nomophobia score was calculated for each participant and transformed into a Z-score in the sample (N=2000). A Z-score lower than -1 was regarded as demonstrating the absence of nomophobia. A Z-score between -1 and the mean was seen as demonstrating a low level of nomophobia. A Z-score above the mean but lower than 1 was taken as demonstrating a mild level of nomophobia. Finally, a Z-score greater than 1 but lower than 2 was recognized as demonstrating a severe level of nomophobia, and a Z-score above 2 was classified as a very severe level of nomophobia. Specifically, there were 15.85% (317/2000), 31.75% (635/2000), 36.95% (739/2000), 13.20% (264/2000), and 2.25% (45/2000) of participants in the without, low, mild, severe, and very severe levels of nomophobia groups, respectively.

### Multivariate Regression Analysis of the Total Scores of Nomophobia and Scores of Various Factors

On the basis of the multivariate regression analysis results shown in Table 6, the NMP-Q total score correlated with daily hours of mobile phone use, gender, and residence, although no statistically significant associations were found with age, grade, and whether or not having a boyfriend or girlfriend.

Identical patterns were found for the total score of the subscale, “not being able to access information.” The total score of the subscale, “giving up convenience,” showed correlations with daily hours of mobile phone use, gender, and grade. “Not being able to communicate” correlated with daily hours of mobile phone use, age, gender, grade, and residence. “Losing connectedness” correlated with daily hours of mobile phone use, gender, grade, and residence.

In conclusion, the daily hours of mobile phone use and gender correlated with all the 4 subscales of the questionnaire, whereas age, grade, residence, and whether or not having a boyfriend or girlfriend only associated with certain subscales. Age had no association with the 4 subscales.

**Table 6:** A multivariate regression analysis on the impact of variables on total scores of the scale and subscales (N=2000).

Model		B		SD		Beta		*t* test (*df*)		*P* value	
**Overall**											
	Constant		30.910		8.597		—^a^		3.595 (6)		<.001
	Number of hours of mobile phone use		3.497		0.383		0.195		9.131 (6)		<.001
	Age (years)		0.726		0.442		0.042		1.643 (6)		.10
	Gender		8.927		0.917		0.209		9.740 (6)		<.001
	Grade		0.520		0.866		0.016		0.600 (6)		.55
	Residence		2.545		0.913		0.060		2.787 (6)		.005
	Having a boyfriend or girlfriend or not		-1.589		1.013		-0.034		-1.569 (6)		.12
**Not being able to access information**											
	Constant		5.279		2.311		—		2.285 (6)		.02
	Number of hours of mobile phone use		0.683		0.103		0.144		6.632 (6)		<.001
	Age (years)		0.144		0.119		0.032		1.210 (6)		.23
	Gender		2.133		0.246		0.189		8.659 (6)		<.001
	Grade		0.273		0.233		0.031		1.171 (6)		.24
	Residence		0.523		0.245		0.046		2.132 (6)		.03
	Having a boyfriend or girlfriend or not		-0.063		0.272		-0.005		-0.233 (6)		.82
**Giving up convenience**											
	Constant		9.967		2.959		—		3.369 (6)		.001
	Number of hours of mobile phone use		1.273		0.132		0.208		9.657 (6)		<.001
	Age (years)		0.225		0.152		0.038		1.482 (6)		.14
	Gender		2.852		0.315		0.195		9.040 (6)		<.001
	Grade		-0.850		0.298		-0.074		-2.850 (6)		.004
	Residence		0.509		0.314		0.035		1.621 (6)		.11
	Having a boyfriend or girlfriend or not		-0.449		0.349		-0.028		-1.286 (6)		.20
**Not being able to communicate**											
	Constant		9.000		2.552		—		3.527 (6)		<.001
	Number of hours of mobile phone use		0.463		0.114		0.089		4.069 (6)		<.001
	Age (years)		0.305		0.131		0.062		2.329 (6)		.02
	Gender		2.169		0.272		0.176		7.974 (6)		<.001
	Grade		-0.882		0.257		-0.091		-3.430 (6)		.001
	Residence		0.671		0.271		0.054		2.474 (6)		.01
	Having a boyfriend or girlfriend or not		-0.568		0.301		-0.042		-1.888 (6)		.06
**Losing connectedness**											
	Constant		6.663		2.905		—		2.294 (6)		.02
	Number of hours of mobile phone use		1.079		0.129		0.179		8.339 (6)		<.001
	Age (years)		0.052		0.149		0.009		0.347 (6)		.73
	Gender		1.774		0.310		0.123		5.727 (6)		<.001
	Grade		1.979		0.293		0.175		6.761 (6)		<.001
	Residence		0.841		0.309		0.058		2.727 (6)		.006
	Having a boyfriend or girlfriend or not		-0.510		0.342		-0.032		-1.489 (6)		.14

^a^Not available.

## Discussion

### Principal Findings

This study translated NMP-Q, developed by Yildirim and Correia [12], into Chinese and tested its psychometric properties among a large number of Chinese college students to investigate whether the Chinese NMP-Q can be applied to college students in China. Our findings supported a structure comprising 18 items falling into 4 factors, which is identical to the structure of the original version of the measure; items 14 and 15 were deleted because of double loadings and because their Chinese meanings are similar to item 13 in the understanding of college students in northern China. Construct validity, discriminant validity, and reliability analysis confirmed that the scale is reliable and valid for assessing nomophobic behavior in Chinese college students.

To the best of our knowledge, our results differ from those of Jianling Ma and Chang Liu in China [15]. One possible reason might be the cultural, territorial, and sample differences. China is a vast country divided into north and south regions, and people’s personalities are different; the southern people are more sensitive than the northern people. The sample of our survey was from northern China, whereas the sample that Jianling Ma and Chang Liu surveyed was from southern China. Therefore, the understanding of items 13, 14, and 15 may be different. The other reason is the sample size (2000 students instead of 966). Therefore, our results may be more appropriate for college students in northern China. The original scale has already been validated in Spain [14], Iran [13], and Italy [1], and in these cases, the content and structural validity of the scale were supported. The Spanish version of NMP-Q showed 4 factors consistent with the original scale. However, in the Persian version, although the 4-factor model was also supported, items 9 and 14 were deleted because of low factor loadings [13]. The Italian version of NMP-Q showed 3 factors [1]. One possible reason might be the cultural and sample differences: although we selected college students, the Persian version used teenagers aged between 13 and 19 years. The Italian version surveyed 403 people, and we surveyed 2000 people, and the people we surveyed were younger than those in the Italian sample (mean 19.07, SD 1.25 years vs mean 27.91, SD 8.63 years, respectively). Therefore, the results are different.

The total scores, both of the scales and all subscales, correlated with the daily hours of mobile phone use and gender, which confirmed the validation of the Chinese version of NMP-Q. Another interesting finding of this study is that gender had a significant influence on nomophobia; specifically, female students were more negatively affected by being without a mobile phone compared with their male counterparts. Our present data are in line with previous studies showing that females were at greater risk of mobile phone addiction [21-23], which may be related to women’s stronger desire for social relationships [21]. In fact, the association between gender and problematic use of mobile phones is quite complex, and, to date, the investigation regarding the effect of gender on nomophobia has produced conflicting results [1,21,24,25]. The discrepancy among studies, including ours, is difficult to understand and might be related to the difference in ethnicity and subgroup of the population included in different studies. In addition, the differential interpretation of the item content and factor structures between genders because of different brain structures might also have an impact on the results [13,26]; this issue warrants further exploration.

Several limitations should be taken into account when interpreting the findings in this study. First, the sample was conveniently obtained; therefore, there is a lack of representativeness. Furthermore, only college students were included in the study. Hence, our results should be generalized with caution. Second, bias was inevitable because of the self-reporting nature of this investigation. Third, data regarding other psychiatric disorders, such as depression, anxiety, and obsessive-compulsive disorders, have not been taken into account, and these may become underlying confounding factors. Besides, because of a dearth of research on this topic, it is difficult to have a proper discussion involving a comparison with the findings of previous studies. Nevertheless, this concurrently highlights the novelty, fun, and readability of this study.

### Conclusions

Nomophobia is an emerging psychological disorder related to problematic mobile phone use and is increasingly studied by health care researchers. Research on this condition might have greater significance for China, as it has become the largest mobile phone handset consumer market in the world. The Chinese version of this instrument, supporting a 4-factor structure, turned out to be reliable; therefore, it can be employed for investigating nomophobia in the Chinese society. Future research should be encouraged to examine the psychometric properties of this translated NMP-Q across different groups in China, to determine underlying comorbidities, and to explore the relationship of nomophobia with other technopathy. In addition, the underlying predictors of nomophobia should be further identified.

## Appendix

Multimedia Appendix 1 Chinese version of the Nomophobia Questionnaire.

## Abbreviations

CFA: confirmatory factor analysis
EFA: exploratory factor analysis
KMO: Kaiser-Meyer-Olkin
NMP-Q: Nomophobia Questionnaire
PCA: principal component analysis
