# Supplementary material for: Translation of the Chinese Version of the Nomophobia Questionnaire and Its Validation Among College Students: Factor Analysis
Source: JMIR Mhealth Uhealth. 2020 Mar 13;8(3):e13561. doi: 10.2196/13561 (PMC7101502; doi:10.2196/13561)
Supplement: Multimedia Appendix 1 [file mhealth_v8i3e13561_app1.pdf]

Multimedia Appendix 1

Chinese version of Nomophobia Questionnaire

1.如果不能一直从手机获取资讯，我会感到不适。
2.如果我想要用手机查资料，却不能用手机时，我会很生气。
3.如果我不能用手机查新闻（如，实事、天气等），我会感到不安。
4.如果我想使用手机和/或它的某项功能，却不能使用时，我会感到烦恼。
5.如果手机快没电了，我会感到不安。
6.如果话费或流量快用完了，我会感到不安。
7.如果我的手机没有数据信号或连不上 Wi-Fi，我会反复查看它是否搜到信号或连上 Wi-Fi。
8.如果我不能使用手机，我怕会遇到麻烦。
9.每隔一段时间，我都会想要拿出手机看一下。
10.如果手机不在身边，我担心不能立即与家人朋友取得联系。
11.如果手机不在身边，我担心家人朋友找不到我。
12.如果手机不在身边，我担心不能接到电话或短信。
13.如果手机不在身边，我会因不能联系家人朋友而焦虑。
14.如果手机不在身边，“是否有人联系我”这种不确定感令我紧张不安。
15.如果手机不在身边，我会因和家人朋友的日常联系被打断而担忧。
16.如果手机不在身边，我会因不能登录社交账户而紧张。
17.如果手机不在身边，我会因接收不到最新的社会媒体和网络热点而感到不适。
18.如果手机不在身边，我会因不能接受到在线更新的通知而感到不自在。
19.如果手机不在身边，我会因不能查看电子邮件而不安。
20.如果手机不在身边，我会因为不知道做什么感到怪怪的。
